# A giant angiomyolipoma arising from the hepatic round ligament: a case report and literature review

**DOI:** 10.3389/fonc.2025.1551361

**Published:** 2025-06-26

**Authors:** Hai-Bo Zheng, Xiang-Qian Wang, Xian-Ni Liu, Xiu-Min Han, Hai-Hong Zhu

**Affiliations:** ^1^ Department of Hepatobiliary Surgery, Qinghai Provincial People’s Hospital, Xining, China; ^2^ Department of Pathology, Qinghai Provincial People’s Hospital, Xining, China

**Keywords:** angiomyolipoma, the round ligament of the liver, tumor, surgery, histological

## Abstract

Angiomyolipoma (AML) is a type of tumor of mesenchymal origin that is most commonly found in the kidney, although it has been found in the liver in rare cases. Herein, we document the first known instance of an AML identified within the liver’s round ligament. A 22-year-old woman presented with a 41-month history of a painless abdominal mass that gradually enlarged. She was misdiagnosed with primary hepatic carcinoma 34 months prior at another hospital and received transcatheter arterial chemoembolization (TACE). After failure of TACE, computed tomography (CT) indicated possible malignant neoplasms. In our hospital, an abdominal CT scan revealed a large mass occupying the right hypochondrium but no evidence of metastatic disease. Consequently, the decision was made to proceed with surgery. During laparotomy, a large, well-defined tumor was discovered, which, surprisingly, was attached to the liver’s round ligament and isolated from any other intra-abdominal structures. The tumor was excised with ease, and monobloc resection and macroscopically total removal were achieved. Histopathological examination confirmed that the tumor was an AML originating from the liver’s round ligament. Postoperative care did not include adjuvant therapy. The patient remains alive and free from any indications or symptoms of recurrence 46 months postsurgery. This case represents the first documented instance of an AML developing from the liver’s round ligament and underscores the diagnostic challenges posed by such tumors. Although uncommon, AML diagnoses should be considered to facilitate early detection and complete surgical excision, which are pivotal for optimal patient outcomes.

## 1 Introduction

Angiomyolipoma (AML) is a type of tumor of mesenchymal origin that is most commonly found in the kidney, but AMLs have been found in the liver in rare circumstances. Approximately 600 AML cases have been reported in the literature in the last 4 decades (1). Most patients with hepatic angiomyolipoma (HAML) lack typical clinical manifestations and specificity in laboratory examinations. Moreover, due to the different proportions of each component of HAMLs, preoperative imaging findings vary greatly, resulting in a high misdiagnosis rate and difficulty in preoperative diagnosis (2). As a rare liver tumor, HAML is generally considered a benign tumor, but studies have shown that it has a potential risk of recurrence and metastasis. A retrospective study by Klompenhouwer et al. (3) reported that the postoperative recurrence rate of HAML was 2.4% and the mortality rate was 0.8%. Given that HAMLs have a certain risk of malignancy and some HAMLs are difficult to distinguish from malignant tumors before surgery, simple conservative treatment may miss malignant tumors, and most scholars advocate early surgical treatment at present (4). This case represents the first documented instance in the medical literature of an AML originating from the round ligament of the liver.

## 2 Case presentation

**Figure 1:**
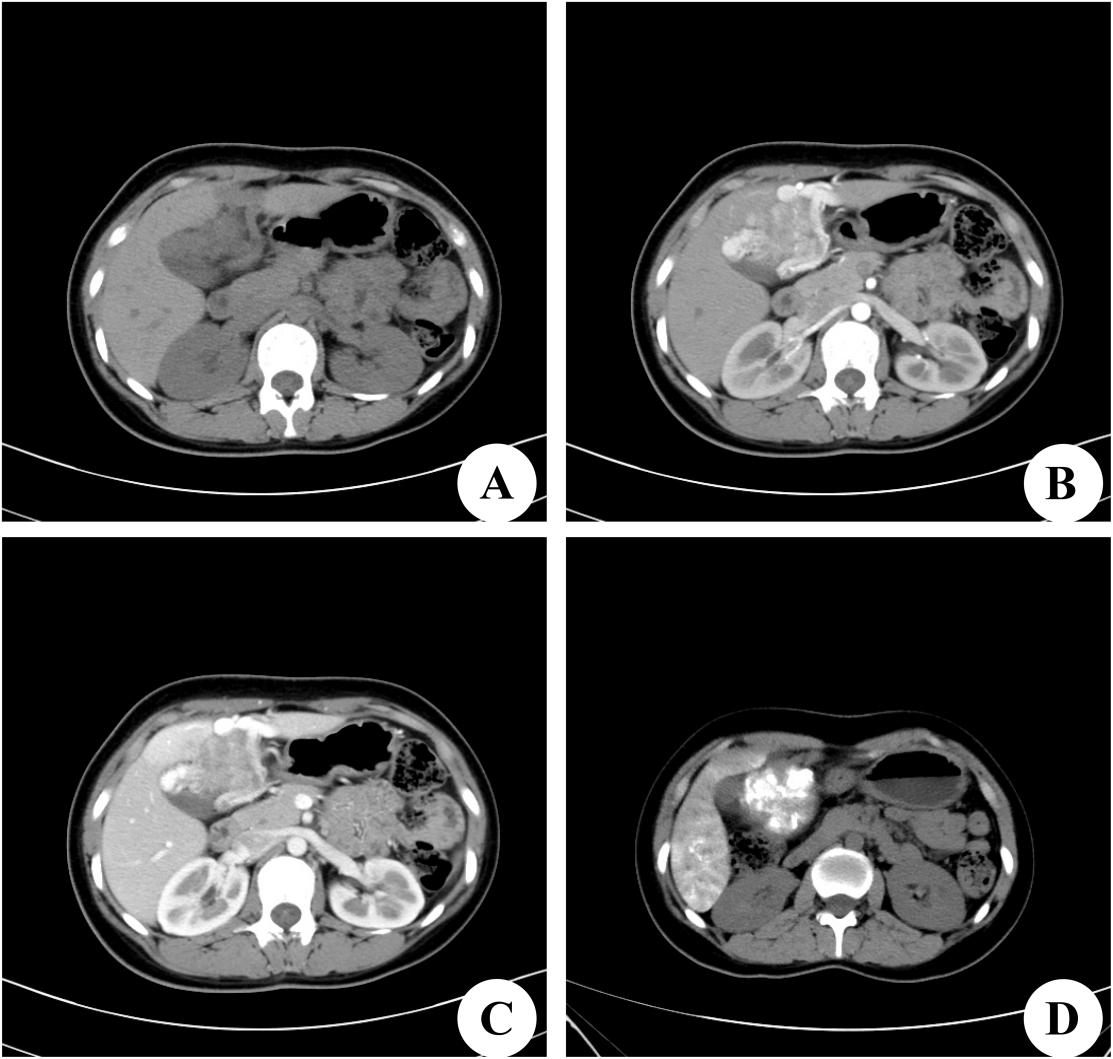
Computed tomography (CT) scan of the first hospital. **(A)** Preoperative CT plain scan. **(B)** On dynamic CT in the arterial phase, there were strongly enhancing areas in the tumor. **(C)** CT scan in the portal venous phase. **(D)** On postoperative CT scan, there is Iodine oil deposition in the center of the tumor.

**Figure 2:**
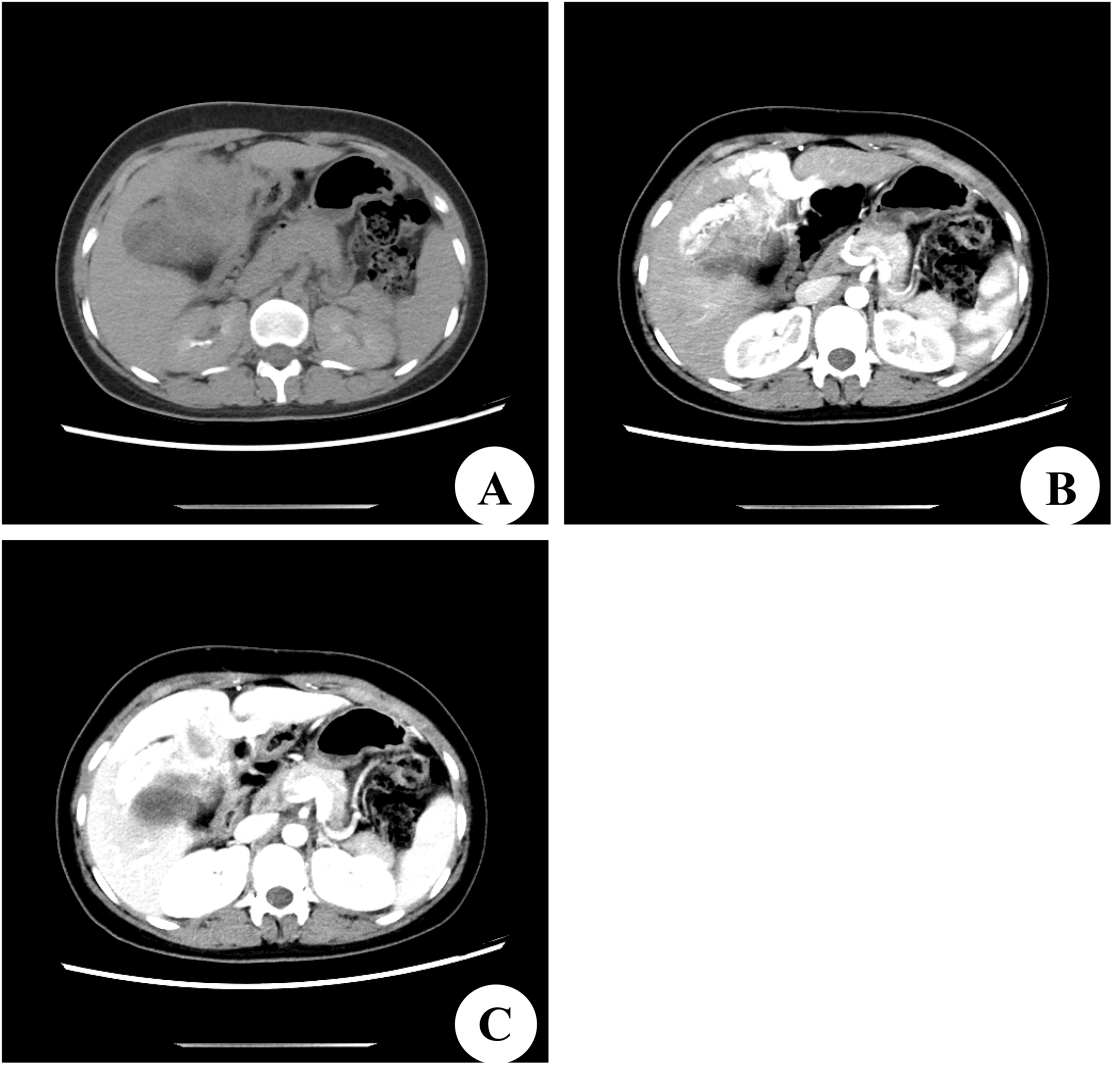
Computed tomography (CT) scan of our hospital. **(A)** Preoperative CT plain scan. **(B)** CT scan in the arterial phase. **(C)** CT scan in the portal venous phase.

A 22-year-old female patient with a right subcostal mass was admitted to the first hospital. An abdominal CT scan revealed that a large mass with an abnormal density shadow measuring 90×50 mm in the abdominal cavity below the liver was significantly enhanced, and necrosis was visible in the lesion, which highly suggested the possibility of malignant neoplasms in the abdomen (**Figures 1A–C**). The hospital staff considered the diagnosis of primary liver cancer and provided TACE treatment (**Figure 1D**). The patient was re-examined in the outpatient department of the hospital after discharge. The results of the third re-examination indicated that the mass was larger than before. Approximately three years later, the patient was transferred to our hospital due to the progressive growth of a mass located below the right costal margin. This mass had been observed for 41 months without any notable alterations in pain levels or tenderness. The patient’s overall health remained stable during this time. No relevant family or personal medical history had contributed to the condition. The patient had a World Health Organization performance status (PS) of 0, indicating no impairment in daily activities. The liver, pancreatic, and kidney functions of the patient were all within normal ranges. A biopsy guided by ultrasound was conducted, and the histological analysis revealed the presence of scattered lymphocytes within the cyst fluid. A subsequent CT scan revealed a solid and cystic mass measuring 120×100 mm and situated beneath the abdominal wall. This mass caused displacement of both the left and right lobes of the liver but remained separate from them; thus, the origin of the mass was likely mesenchymal neoplasms (**Figures 2A–C**). On the basis of these findings, the decision was made to proceed with surgical intervention.

**Figure 3:**
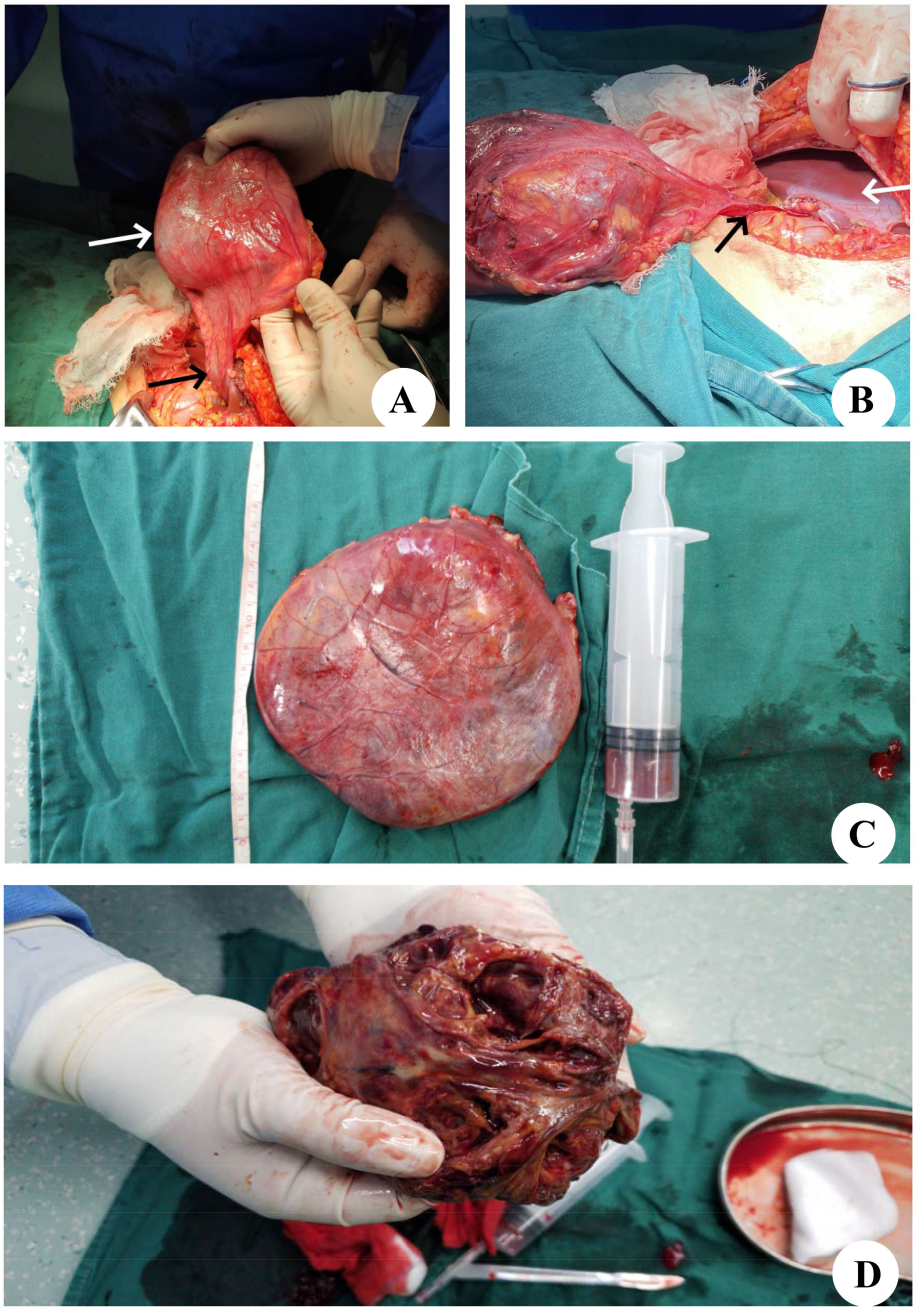
Intraoperative findings and histological aspects. **(A)** The tumor (white arrow) is appended to the round ligament of the liver (black arrow). **(B)** The tumor is free from any other intra-abdominal contact. The black arrow shows the round ligament of the liver, and the white arrow shows the liver parenchyma. **(C)** After complete resection of the tumor. **(D)** Macroscopic aspect on cut section showed a firm, gray-white tumor.

**Figure 4:**
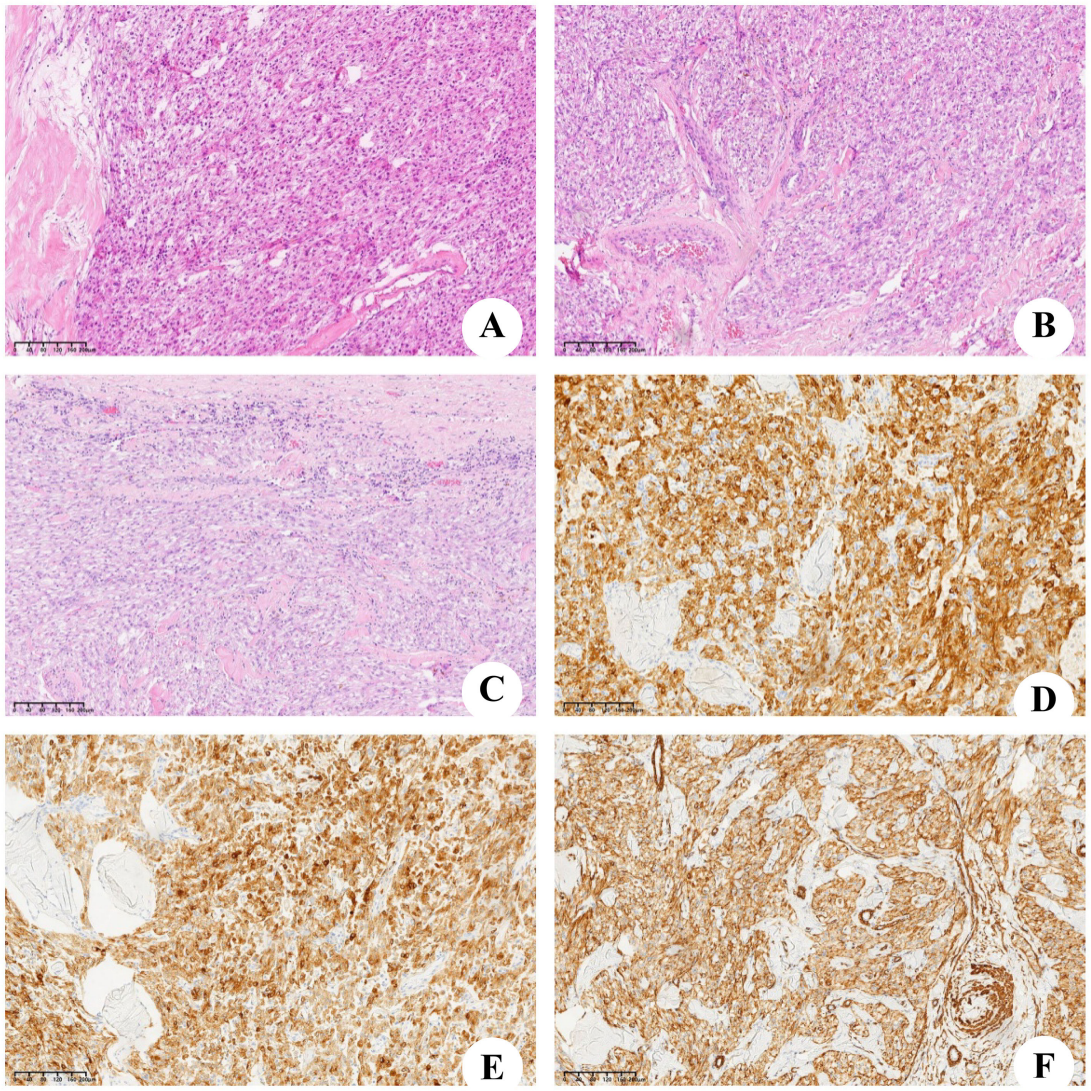
Microscopic and immunohistochemical findings. **(A–C)** Microscopic aspect, the tumor was consist of lots of spindle cells and little adipocyte. **(D)** Immunostain for Melan-A. **(E)** Immunostain for HMB45. **(F)** Immunostain for smooth muscle actin (SMA).

During laparotomy, a large and well-defined tumor was discovered, which was unexpectedly attached to the round ligament of the liver (**Figure 3A**) and exhibited a ‘caput medusae’ pattern of vascularization. The tumor was isolated from other intra-abdominal structures, particularly the liver parenchyma (**Figure 3B**), and was easily excised, requiring no additional dissection. The removal was performed en bloc and was macroscopically complete. The excised tumor measured 120×90 mm and weighed 2,030 g (**Figure 3C**). Upon sectioning, the tumor presented a fleshy consistency with gray–white coloration (**Figure 3D**). Unlike the initial findings from the microbiopsy, the histological examination of the surgically removed specimen revealed that the cells were arranged in a cord-like arrangement, and most of the cells were epithelioid, with no clear nuclear mitosis or necrosis; blood sinusoid spaces or bleeding between the cells, hyalinoid degeneration of the vascular wall of the interstitium, and little fat around the mass were observed (**Figures 4A–C**). Immunohistochemistry (IHC) analysis confirmed the expression of Melan-A, HMB-45 and SMA by the tumor cells (**Figures 4D–F**). The final diagnosis confirmed an AML originating from the round ligament of the liver. The patient’s recovery after surgery was smooth, with no complications reported. Adjuvant therapy was not administered as part of the treatment plan. A schedule for regular clinical and radiological check-ups was established to monitor the patient’s condition. At the 46-month follow-up visit, the patient was alive, maintained an excellent performance status (PS), and showed no indications or symptoms of disease recurrence.

## 3 Discussion

The etiology and pathogenesis of AML are not entirely clear. Currently, the tumor cells are believed to originate from perivascular epithelioid cells (PECs) associated with perivascular epithelioid tumors (PEComas) (5) and have multidirectional differentiation potential. As a benign mesenchymal tumor, AML can differentiate into vascular smooth muscle and epithelial cells and expresses melanoma-related antibodies (6–8). HAML may be associated with tuberous sclerosis (TSC) gene mutations (9, 10).

The round ligament, also known as the ligamentum teres, is a fibrous structure resulting from the involution of the umbilical vein after birth. It serves as an anatomical divider, separating the left lobe of the liver into medial and lateral sections. Tumors originating in the round ligament are exceptionally rare, and the ligamentum teres hepatis (LTH) is one of the least common locations for primary tumor development. A review of the literature revealed that both benign and malignant tumors can develop in the LTH, and most of these tumors are metastases originating from gastrointestinal cancers, particularly mucinous appendiceal neoplasms, as well as peritoneal mesothelioma (11). In rarer cases, they may also arise from breast cancer or hepatocellular carcinoma (12, 13). Instances of primary mesenchymal tumors have also been documented, including sarcomas (malignant fibrous histiocytoma, leiomyosarcoma) (14), and benign tumors such as lipomas, lymphangiomas, stromal tumors (15) and SFTs (16). To the best of our knowledge, there are only 10 reported cases of PEComas of the falciform ligament/ligamentum teres (17–21). To date, there have been no reported cases of AML originating from the round ligament of the liver.

HAML is more common in young and middle-aged females. Klompenhouwer et al. (22) researched and analyzed 292 cases of HAML and reported that the proportion of males to females was approximately 1:3. HAML was first reported by Ishak (23) in 1976 and in China by Wen-Ming Cong (24) in 1992. Most patients had no obvious clinical symptoms, and a small number of them had atypical symptoms such as dull pain or abdominal distension with increasing tumor volume. Imaging examination lacks specificity, and distinguishing HAML from liver cancer or other benign tumors is difficult sometimes. The correct diagnosis depends on pathology and IHC. Under a microscope, a tumor is composed of three components: blood vessels, smooth muscle cells and fat cells. The initial histopathological examination suggested a diagnosis of either leiomyoma or fibroma, but these possibilities were later ruled out by our team. The positive expression of Melan-A, HMB-45 and SMA in tumor cells by IHC is reliable evidence for the diagnosis of HAML (25–27). Ki-67, an index of cell proliferation, is positively correlated with the degree of malignancy of the tumor. IHC of the tumor of this patient revealed a Ki-67 value of 5%, indicating that the tumor was in a state of low proliferation.

HAMLs are typically classified as benign tumors according to their prognosis. However, their clinical progression can be unpredictable, and malignant transformation of HAML is rare, especially in the epithelioid subtype (28). Many experts consider complete surgical resection of the tumor to be the most critical prognostic factor. The AML originating from the hepatic round ligament typically originates within the ligamentum teres and is characterized by relatively low fat content and a weaker association with tuberous sclerosis complex (TSC). In contrast, hepatic AMLs can occur in any location within the liver parenchyma, exhibits more variable fat content, and is associated with TSC in approximately 5%-10% of cases. In this particular case, the tumor’s “fortunate” location in the round ligament allowed straightforward and total removal, with the only adverse factor being its considerable size. Surgical removal is the preferred treatment for AMLs, and when the tumor is fully excised, the 5-year survival rate approaches 100%.

## 4 Conclusion

This case represents the first documented instance in the medical literature of an AML originating from the round ligament of the liver. This case highlights the diagnostic challenges associated with such tumors. Despite their rarity, this diagnosis of AML should be considered by physicians to allow for early diagnosis and complete surgical removal, which offer the highest likelihood of a successful recovery.

## Data Availability

The original contributions presented in the study are included in the article/supplementary material. Further inquiries can be directed to the corresponding authors.
